# Optimal Control of HIV Dynamic Using Embedding Method

**DOI:** 10.1155/2011/674318

**Published:** 2011-04-18

**Authors:** H. Zarei, A. V. Kamyad, M. H. Farahi

**Affiliations:** Department of Applied Mathematics, Ferdowsi University of Mashhad, Mashhad 91775, Iran

## Abstract

This present study proposes an optimal control problem, with the final goal of implementing an optimal treatment protocol which could maximize the survival time of patients and minimize the cost of drug utilizing a system of ordinary differential equations which describes the interaction of the immune system with the human immunodeficiency virus (HIV). Optimal control problem transfers into a modified problem in measure space using an embedding method in which the existence of optimal solution is guaranteed by compactness of the space. Then the metamorphosed problem is approximated by a linear programming (LP) problem, and by solving this LP problem a suboptimal piecewise constant control function, which is more practical from the clinical viewpoint, is achieved. The comparison between the immune system dynamics in treated and untreated patients is introduced. Finally, the relationships between the healthy cells and virus are shown.

## 1. Introduction

 Human immunodeficiency virus infects CD4+ T-cells, which are an important part of the human immune system, and other target cells. The infected cells produce a large number of viruses. Medical treatments for HIV have greatly improved during the last two decades. Highly active antiretroviral therapy (HAART) allows for the effective suppression of HIV-infected individuals and prolongs the time before the onset of acquired immune deficiency syndrome (AIDS) for years or even decades and increases life expectancy and quality to the patient. But antiretroviral therapy cannot eradicate HIV from infected patients because of the long-lived infected cells and sites within the body where drugs may not achieve effective levels [1–3]. HAART contains two major types of anti-HIV drugs: reverse transcriptase inhibitors (RTI), and protease inhibitors (PI). Reverse transcriptase inhibitors prevent HIV from infecting cells by blocking the integration of the HIV viral code into the host cell genome while protease inhibitors prevent infected cells from replication of infectious virus particles, and can reduce and maintain viral load below the limit of detection in many patients. Moreover, treatment with either type of drugs can also increase the CD4+ T-cell counts that are target cells for HIV. 

Many of the host-pathogen interaction mechanisms during HIV infection and progression to AIDS are still unknown. Mathematical modeling of HIV infection is of interest to the medical community as no adequate animal models exist to test the efficacy of drug regimes. These models can test different assumptions and provide new insights into questions that are difficult to answer by clinical or experimental studies. A number of mathematical models have been formulated to describe various aspects of the interaction of HIV with healthy cells. Some of these models are addressed in [4]. The basic model of HIV infection is presented by Wodarz and Nowak [5], which contains three state variables: healthy CD4+ T-cells, infected CD4+ T-cells, and concentration of free virus. Their model has been modified to offer important theoretical insights into immune control of the virus, based on treatment strategies, while maintaining a simple structure [6]. Furthermore, this modified model has been developed to guess the natural evolution of HIV infection, as qualitatively described in several clinical studies [7]. 

 Some authors have used mathematical models for HIV infection in conjunction with control theory to achieve appropriate goals. For example, these goals may include maximizing the level of healthy CD4+ T-cells and minimizing the cost of treatment [8–11], maximizing the level of healthy CD4+ T-cells while minimizing both the cost of treatment and viral load [12], minimizing both the HIV population and systemic costs to body while maximizing immune response [13, 14], and maximizing both the healthy CD4+ T-cell counts and immune response while minimizing the cost of treatment [15], maximizing the healthy CD4+ T-cell counts and minimizing both the side effects and drug resistance [16].

 The papers [17–21] consider only RTI medication while the papers [22, 23] consider only PIs. In [24–27], all effects of a HAART medication are combined to one control variable in the model. In [28–32], dynamical multidrug therapies based on RTIs and PIs are designed.

 In this paper, a mathematical model of HIV dynamic is considered that includes the effect of antiretroviral therapy, and an analysis of optimal control is performed regarding appropriate goals.

 The paper is organized as follows: in Section 2, the underlying HIV mathematical model is described. Our formulation of the control problem, which attempts to prolong the survival time of patient as long as possible, is described in Section 3. Approximating the obtained optimal control problem by an LP problem is the subject of Section 4. Numerical results obtained from solving the LP problem are presented in Section 5. Finally, Section 6 is assigned to concluding remarks.

## 2. Presentation of a Working Model

In this paper, the pathological behavior of HIV is considered which is modeled with the simplified version of a system of ordinary differential equations (ODEs) as described in [17]. This model, which is consistent with clinical data, is given as follows:

(1) $$\begin{array}{c} \frac{d\overset{̅}{P}\left(t\right)}{dt}=I_{\overset{̅}{P}}+\beta \left(P_{0}-P\left(t\right)\right)-{\overset{̅}{\tau }}_{p}\overset{̅}{P}\left(t\right)-{\overset{̅}{c}}_{P}a\left(t\right)C\left(t\right)\overset{̅}{P}\left(t\right), \end{array}$$

(2) $$\begin{array}{c} \frac{dP\left(t\right)}{dt}={\overset{̅}{\tau }}_{P}\overset{̅}{P}\left(t\right)-\tau_{P}P\left(t\right)-c_{P}a\left(t\right)C\left(t\right)P\left(t\right), \end{array}$$

(3) $$\begin{array}{c} \frac{da\left(t\right)}{dt}=a\left(t\right)\left(\kappa \theta -\gamma C\left(t\right)\right), \end{array}$$

(4) $$\begin{array}{c} \frac{dC\left(t\right)}{dt}=a\left(t\right)\left(\varepsilon I_{C}+\alpha C\left(t\right)\right){\left(\frac{P\left(t\right)}{P_{0}}\right)}^{\upsilon }-\tau_{C}C\left(t\right). \end{array}$$

Most of the terms in this model have straightforward interpretations. $\overset{̅}{P}(\cdot )$ and *P*(·) denote the amounts of immature CD4+ T-cells and mature ones, respectively. The term *a*(·) indicates HIV particles, and *C*(·) designates cytotoxic T-cells specific for HIV (CTLs) as a function of time. Here, $I_{\overset{̅}{P}}$ is the constant rate that $\overset{̅}{P}$ cells are produced, ${\overset{̅}{\tau }}_{p}$ is the rate of maturation of $\overset{̅}{P}$ cells into *P* cells, and *τ*_*p*_ is the rate of natural death of *P* cells. Furthermore, *β* is the amplifying coefficient of the linear feedback effect of *P* cells decrease on the influx of $\overset{̅}{P}$ cells at time *t*. Free virus particles *a*(*t*) eliminate $\overset{̅}{P}(\cdot )$ cells at a rate proportional to ${\overset{̅}{c}}_{P}a\left(t\right)C\left(t\right)\overset{̅}{P}\left(t\right)$ at time *t*. Similarly, *c*_*P*_ *a*(*t*)*C*(*t*)*P*(*t*) is the rate of elimination of *P* cells. The term *θ* characterizes the growth rate of HIV particles, and *γ* is the rate of inactivation of HIV products mediated by cytotoxic *C* cells. *I*_*C*_ is the influx of *C* cell precursors, *ε* is their maturation rate, *α* is the proliferation rate of *C* cells under the antigenic stimulation by HIV products, and *τ*_*C*_ is their natural death rate. Helper T-cells effect on maturation and proliferation of *C* cells is expressed by the ratio *P*(*t*)/*P*_0_, and *ν* is introduced to characterize the intensity of this helper effect. Chemotherapeutic agent was simulated by decreasing the value *κ*, that is, the HIV proliferation rate. Lower value for *κ* corresponds to higher RTI-drug doses.

## 3. Optimal Control Formulation

 In this section, we formulate an optimal control problem that identifies the inhibition parameter *κ* in (3), with a function of the control variable. In particular, we will replace the parameter *κ* with the function 1 − *u*(*t*). This choice then identifies the control variable *u*(*t*) with the rate of inhibition of virus reproduction, which is modeled as a simple function of drug dosage.

In clinical practice, the following guidelines are used typically.

- Antiretroviral therapy is initiated at *t*_0_, the time at which the CD4+ T-cell count falls below 350 cells/*μ*L.
- The transition from HIV to AIDS is marked by a CD4+ T-cell count below 200 cells/*μ*L.
- A person is said to have full-blown AIDS when his/her CD4+ T-cell count falls below CD4_crit_^+^, typically around of 50 cells/*μ*L.

This paper aims to propose a drug regimen that delays the onset of full-blown AIDS and prolongs survival as much as possible, while one is going to minimize the drug costs. This can be modeled as follows.

Assume that the onset of full-blown AIDS occurs after time *t*_*f*_. Hence, we should have

(5) $$P\left(t\right)\geq \text{CD}4_{\text{crit}}^{+},\;t\in \left[t_{0},t_{f}\right],\;\;P\left(t_{f}\right)=\text{CD}4_{\text{crit}}^{+}.$$

A problem arising from the use of most chemotherapies is the multiple and sometimes harmful side effects, as well as the ineffectiveness of treatment after a certain time due to the capability of the virus to mutate and become resistant to the treatment. Global effects of these phenomena can be considered by imposing limited treatment interval [22], that is, treatment lasting for a given period from time *t*_0_ to *t*_0_ + *η*. Therefore, the support of the control function *u*(·) must be in the treatment interval

(6) $$\mathrm{supp}\;\;u\subseteq \left[t_{0},t_{0}+\eta \right].$$

Here, we follow [8, 22] in assuming that the costs of the treatment is proportional to *u* ^2^(*t*) at time *t*. Therefore, the overall cost of the treatment is ∫_*t*_0__^*t*_*f*_^ *u* ^2^(*t*)*dt*. So, the following functional should be maximized:

(7) $$\sigma \left(t_{f},u\right)=t_{f}-\lambda \overset{t_{f}}{\underset{t_{0}}{\int }}u^{2}\left(t\right)dt.$$

Parameter *λ* is used to set the relative importance between maximizing the survival time *t*_*f*_ and minimizing the systemic cost to the body. Setting $\overset{̅}{P}=x_{1}$, *P* = *x*_2_, *a* = *x*_3_, and *C* = *x*_4_, the system of differential equations (1)–(4) can be represented in a generalized form as

(8) $$\begin{array}{cc} \dot{x}\left(t\right) & =g\left(t,x\left(t\right),u\left(t\right)\right)\\ & =\left(\begin{array}{c} I_{P}+\beta \left(x_{2}^{0}-x_{2}\right)-{\overset{̅}{\tau }}_{P}x_{1}-{\overset{̅}{c}}_{P}x_{3}x_{4}x_{1}\\ {\overset{̅}{\tau }}_{P}x_{1}-\tau_{P}x_{2}-c_{P}x_{3}x_{4}x_{2}\\ x_{3}\left(\left(1-u\right)\theta -\gamma x_{4}\right)\\ \left(\varepsilon I_{C}+\alpha x_{4}\right)x_{3}{\left(\frac{x_{2}}{x_{2}^{0}}\right)}^{\upsilon }-\tau_{C}x_{4} \end{array}\right),\;x\left(0\right)=x_{0}. \end{array}$$

Assume that *K* denotes the set of all measurable control functions *u*(·)∈[0, 1], where *u*(·) satisfies (6), and the corresponding solution of (8) at final time *t*_*f*_ satisfies (5). Therefore, we are seeking for u*(·) ∈ *K* such that

(9) $$\sigma \left(t_{f},u\right)\leq \sigma \left(t_{f}^{*},u^{*}\right),\;\forall u\in K.$$

Setting *f*_0_(*t*, *x*(*t*), *u*(*t*)) = 1 − *λu* ^2^(*t*), then the optimal drug regimen problem, while ignoring *t*_0_, can be represented as:

(10) $$\underset{t_{f},\;u\;\in \;K}{\max\;}\;\overset{t_{f}}{\underset{t_{0}}{\int }}f_{0}\left(t,x\left(t\right),u\left(t\right)\right)dt\;$$

subject to

(11) $$\begin{array}{c} \dot{x}=g\left(t,x\left(t\right),u\left(t\right)\right), \end{array}$$

(12) $$\begin{array}{c} x\left(t_{0}\right)=x_{t_{0}},\;\;x_{2}\left(t_{f}\right)=\text{CD}4_{\text{crit}}^{+}, \end{array}$$

(13) $$\begin{array}{c} x_{2}\left(t\right)\geq \text{CD}4_{\text{crit}}^{+},\;t\in \left[t_{0},t_{f}\right] \end{array}.$$

This optimal control problem is referred to as OCP. Some problems may arise in the quest of solving OCP. The set *K* may be empty. If *K* is not empty, the functional measuring the performance of the system may not achieve its maximum in the set *K*. In order to overcome these difficulties, in the next section we transfer the OCP into a modified problem in measure space.

## 4. Approximation of OCP by Linear Programming Problem

Using measure theory for solving optimal control problems based on the idea of Young [33], which was applied for the first time by Wilson and Rubio [34], has been theoretically established by Rubio in [35]. Then, the method has been extended for approximating the time optimal problems by an LP model [36]. Here, this approach is used.

### 4.1. Functional Space

We assume that the state variables *x*(·) and the control input *u*(·), respectively, get their values in the compact sets *A* = *A*_1_ × *A*_2_ × *A*_3_ × *A*_4_ ⊂ *ℜ* ^4^ and *U* ⊂ *ℜ*. Set *J* = [*t*_0_, *t*_*f*_]. Here, we are going to derive weak forms for (11)–(13).

**Definition 1**

A triple *p* = [*t*_*f*_, *x*, *u*] is said to be admissible if the following conditions hold.

- The vector function *x*(·) is absolutely continuous and belongs to *A* for all *t* ∈ *J*.
- The function *u*(·) takes its values in the set *U* and is Lebesgue measurable on *J*.
- *p* satisfies in (11)–(13), on *J* ^0^, that is, the interior set of *J*.

It is assumed that the set of all admissible triples is nonempty and denotes it by *W*. Let *p* be an admissible triple, *B* be an open ball in *ℜ* ^5^ containing *J* × *A*, and let *C*′(*B*) be the space of all real-valued continuous differentiable functions on it. Let *φ* ∈ *C*′(*B*), and define *φ* ^*g*^ as follows:

(14) $$\varphi^{g}\left(t,x\left(t\right),u\left(t\right)\right)=\varphi_{x}\left(t,x\left(t\right)\right)\cdot g\left(t,x\left(t\right),u\left(t\right)\right)+\varphi_{t}\left(t,x\left(t\right)\right)$$

for each [*t*, *x*(*t*), *u*(*t*)] ∈ *Ω*, where *Ω* = *J* × *A* × *U*. The function *φ* ^*g*^ is in the space *C*(*Ω*), the set of all continuous functions on the compact set *Ω*. Since *p* = [*t*_*f*_, *x*, *u*] is an admissible triple, we have

(15) $$\begin{array}{cc} \overset{t_{f}}{\underset{t_{0}}{\int }}\varphi^{g}\left(t,\xi \left(t\right),u\left(t\right)\right)dt & =\overset{t_{f}}{\underset{t_{0}}{\int }}\varphi_{x}\left(t,x\left(t\right)\right)\cdot \dot{x}\left(t\right)+\varphi_{t}\left(t,x\left(t\right)\right)dt\\ & =\varphi \left(t_{f},x\left(t_{f}\right)\right)-\varphi \left(t_{0},x\left(t_{0}\right)\right)=\Delta \varphi , \end{array}$$

for all *φ* ∈ *C*′(*B*). Let *D*(*J* ^0^) be the space of all infinitely differentiable real-valued functions with compact support in *J* ^0^. Define

(16) $$\begin{array}{cc} \psi^{\;n}\left(t,x\left(t\right),u\left(t\right)\right)=x_{n}\left(t\right)\psi^{\prime }\left(t\right)+g_{n}\left(t,x\left(t\right),u\left(t\right)\right)\psi \left(t\right), & \\ n=1,2,3,4,\;\;\forall \psi \in D\left(J^{0}\right). & \end{array}$$

Assume *p* = [*t*_*f*_, *x*, *u*] be an admissible triple. Since the function *ψ*(·) has compact support in *J* ^0^, *ψ*(*t*_0_) = *ψ*(*t*_*f*_) = 0. Thus, for *n* = 1,2, 3,4, and for all *ψ* ∈ *D*(*J* ^0^), from (16) and using integration by parts, we have

(17) $$\begin{array}{cc} \overset{t_{f}}{\underset{t_{0}}{\int }}\psi^{n}\left(t,x\left(t\right),u\left(t\right)\right)dt & =\overset{t_{f}}{\underset{\;t_{0}}{\int }}x_{n}\left(t\right)\psi^{\prime }\left(t\right)dt\\ & \;+\overset{t_{f}}{\underset{t_{0}}{\int }}g_{n}\left(t,x\left(t\right),u\left(t\right)\right)\psi \left(t\right)dt\\ & =0. \end{array}$$

Also, by choosing the functions which are dependent only on time, we have

(18) $$\overset{t_{f}}{\underset{t_{0}}{\int }}\vartheta \left(t,x\left(t\right),u\left(t\right)\right)dt=a_{\vartheta },\;\forall \vartheta \in C^{1}\left(\Omega \right),$$

where *C* ^1^(*Ω*) is the space of all functions in *C*(*Ω*) that depend only on time and *a*_*ϑ*_ is the integral of *ϑ*(·) on *J*.

Equations (15), (17), and (18) are the weak forms of (11)–(13). Note that the constraints (12) are considered on the right-hand side of (15) by choosing suitable functions *φ* ∈ *C*′(*B*) which are monomials of *x*_2_. Furthermore, the constraint (13) is considered, by choosing an appropriate set *A*. Now, we consider the following positive linear functional on *C*(*Ω*):

(19) $$\Gamma_{p}:F\to \int_{J}F\left(t,x\left(t\right),u\left(t\right)\right)dt,\;\forall F\in C\left(\Omega \right).$$

**Proposition 1**

Transformation *p* → Γ_*p*_ of admissible triples in *W* into the linear mappings Γ_*p*_ defined in (19) is an injection.

**Proof**

We must show that if *p*_1_ ≠ *p*_2_, then Γ_*p*_1__ ≠ Γ_*p*_2__. Let *p*_*j*_ = [*t*_*f*_*j*__, *x*_*j*_, *u*_*j*_], *j* = 1, 2 be different admissible triples. If *t*_*f*_1__ = *t*_*f*_2__, then there is a subinterval of [*t*_0_, *t*_*f*_1__], say *J*_1_, where *x*_1_(*t*) ≠ *x*_2_(*t*) for each *t* ∈ *J*_1_. A continuous function *F* can be constructed on *Ω* so that the right-hand sides of (19) corresponding to *p*_1_ and *p*_1_ are not equal. For instance, one can make *F* independent of *u*, equal zero for all *t* outside *J*_1_, and such that it is positive on the appropriate portion of *x*_1_(·), and zero on the *x*_2_(·), then the linear functionals are not equal. In other words, if *t*_*f*_1__ ≠ *t*_*f*_2__, then Γ_*p*_1__ and Γ_*p*_2__ have different domains and are not equal.

Thus, from (15), (17), and (18), one can conclude that maximizing the functional (10) over admissible space *W*, changes to the following optimization problem in functional space:

(20) $$\;\underset{p\in W}{\max\;}\;\;\Gamma_{p}\left(f_{0}\right)\;\;$$

subject to

(21) $$\begin{array}{c} \Gamma_{p}\left(\varphi^{g}\right)=\Delta \varphi ,\;\varphi \in C^{\prime }\left(B\right), \end{array}$$

(22) $$\begin{array}{c} \Gamma_{p}\left(\psi^{n}\right)=0,\;n=1,2,3,4,\;\;\psi \in D\left(J^{0}\right), \end{array}$$

(23) $$\begin{array}{c} \Gamma_{p}\left(\vartheta \right)=a_{\vartheta },\;\vartheta \in C^{1}\left(\Omega \right). \end{array}$$

### 4.2. Measure Space

 Let *M* ^+^(*Ω*) denote the space of all positive Radon measures on *Ω*. By the Riesz representation theorem [35], there exists a unique positive Radon measure *μ* on *Ω* such that

(24) $$\begin{array}{cc} \Gamma_{p}\left(F\right) & =\int_{J}F\left(t,x\left(t\right),u\left(t\right)\right)dt\\ & =\int_{\Omega }F\left(t,x,u\right)d\mu \equiv \mu \left(F\right),\;F\in C\left(\Omega \right). \end{array}$$

So, we may change the functional space of the optimization problem to measure space. In other words, the optimization problem (20)–(23) can be converted to the following optimization problem in measure space:

(25) $$\underset{\mu \in M^{+}\left(\Omega \right)}{\text{Maximize}}\;\;\mu \left(f_{0}\right)$$

subject to

(26) $$\begin{array}{c} \mu \left(\varphi^{g}\right)=\Delta \varphi ,\;\varphi \in C^{\prime }\left(B\right), \end{array}$$

(27) $$\begin{array}{c} \mu \left(\psi^{n}\right)=0,\;n=1,2,3,4,\;\;\psi \in D\left(J^{0}\right), \end{array}$$

(28) $$\begin{array}{c} \mu \left(\vartheta \right)=a_{\vartheta },\;\vartheta \in C^{1}\left(\Omega \right). \end{array}$$

We will consider maximization of (25) over the set *Q* of all positive Radon measures on *Ω*, satisfying (26)–(28). The main advantages of considering this measure theoretic form of the problem is the existence of optimal measure in the set *Q* where this point can be studied in a straightforward manner without having to impose conditions such as convexity which may be artificial. 

Define function *I* : *Q* → *R* as *I*(*μ*) = *μ*(*f*_0_). The following theorem guarantees the existence of an optimal solution.

**Theorem 1**

The measure theoretical problem of maximizing (25) with constraints (26)–(28) has an optimal solution, say μ*, where μ* ∈ *Q*.

**Proof**

The so-called constraints (27) and (28) are special cases of (26) [35]. So, the set *Q *can be written as

(29) $$Q=\underset{\varphi \in C^{\prime }(B)}{⋂}\left\{\mu \in M^{+}\left(\Omega \right):\;\mu \left(\varphi^{g}\right)=\Delta \varphi \right\}.$$

Assume that *p* = [*t*_*f*_, *x*, *u*] is an admissible triple. It is well known that the set {*μ* ∈ *M* ^+^(*Ω*) : *μ*(1) = *t*_*f*_ − *t*_0_} is compact in the weak* topology. Furthermore, the set *Q* as intersection of inverse image of closed singleton sets {Δ*φ*} under the continuous functions *μ* → *μ*(*φ* ^*g*^) is also closed. Thus, *Q* is a closed subset of a compact set. This proves the compactness of the set *Q*. Since the functional *I*, mapping the compact set *Q* on the real line, is continuous and thus takes its maximum on the compact set *Q*.

Next, based on analysis in [35], the problem (25)–(28) is approximated by an LP problem, and a triple p* which approximates the action of μ* ∈ *Q* is achieved.

### 4.3. Approximation

The problem (25)–(28) is an infinite-dimensional linear programming problem, and we are mainly interested in approximating it. First, the maximization of *I* is considered not over the set *Q*, but over a subset of it denoted by requiring that only a finite number of constraints (26)–(28) be satisfied. Let {*φ*_*i*_ : *i* = 1,2,…}, {*ψ*_*j*_ : *j* = 1, 2,…}, and {*ϑ*_*s*_ : *s* = 1,2,…} be the sets of total functions, respectively, in *C*′(*B*), *D*(*J* ^0^), and *C* ^1^(*Ω*). The first approximation is completed by choosing finite number of functions *φ*_*i*_s, *ψ*_*j*_s, and *ϑ*_*s*_s. Now we have the following propositions.

**Proposition 2**

Consider the linear program problem consisting of maximizing the function *I* over the set *Q*_*M*_ of measures in *M* ^+^(*Ω*) satisfying:

(30) $$\mu \left(\varphi_{i}^{g}\right)=\Delta \varphi_{i},\;i=1,\ldots ,M.$$

Then, *J*_*M*_ ≡ max _*Q*_*M*__ *I* tends to *J* = max _*Q*_ *I* as *M* → *∞*.

**Proof**

We have *Q*_1_⊇*Q*_2_⊇⋯⊇*Q*_*M*_⊇⋯⊇*Q* and hence, *J*_1_ ≥ *J*_2_ ≥ ⋯≥*J*_*M*_ ≥ ⋯≥*J*. The sequence {*J*_*j*_}_*j*=1_^*∞*^ is nonincreasing and bounded, so, it converges to a number *ζ* such that *ζ* ≥ *J*. We show that *ζ* = *J*. Set *R* ≡ ⋂_*M*=1_^*∞*^ *Q*_*M*_. Then, *R*⊇*Q* and *ζ* ≡ max_*R*_ *I*. It is sufficient to show *R*⊆*Q*. Assume *μ* ∈ *R* and *φ* ∈ *C*′(*B*). Since the linear combinations of the functions {*φ*_*j*_, *j* = 1,2,…} are uniformly dense in *C*′(*B*), there is a sequence $\left\{{\tilde{\varphi }}_{k}\right\}\in \mathrm{span}\;\;\left\{\varphi_{j},j=1,2,\ldots \right\}$, such that ${\tilde{\varphi }}_{k}$ tends to *φ* uniformly as *k* → *∞*. Hence, *S*_1_, *S*_2_, and *S*_3_ tend to zero as *k* → *∞* where $S_{1}=\sup\;\left|\varphi_{x}-{\tilde{\varphi }}_{k_{x}}\right|$, $S_{2}=\sup\;\left|\varphi_{t}-{\tilde{\varphi }}_{k_{t}}\right|$, and $S_{3}=\sup\;\left|\varphi -{\tilde{\varphi }}_{k}\right|$. Since *μ* ∈ *R* and the functional *f* → *μ*(*f*) is linear, $\mu ({{\tilde{\varphi }}_{k}}^{g})=\Delta {\tilde{\varphi }}_{k}$ and

(31) $$\begin{array}{cc} & \left|\mu \left(\varphi^{g}\right)-\Delta \varphi \right|\\ & \;\;=\left|\mu \left(\varphi^{g}\right)-\Delta \varphi -\mu \left({{\tilde{\varphi }}_{k}}^{g}\right)+\Delta {\tilde{\varphi }}_{k}\right|\\ & \;\;=|\int_{\Omega }\{\left[\varphi_{x}\left(t,x\right)-{\tilde{\varphi }}_{k_{x}}\left(t,x\right)\right]g\left(t,x,u\right)\\ & \;\;\;\;\;\;+\left[\varphi_{t}\left(t,x\right)-{\tilde{\varphi }}_{k_{t}}\left(t,x\right)\right]\}d\mu -\left(\Delta \varphi -\Delta {\tilde{\varphi }}_{k}\right)|\\ & \;\;\leq S_{1}\int_{\Omega }\left|g\left(t,x,u\right)\right|d\mu +S_{2}\int_{\Omega }d\mu +2S_{3}. \end{array}$$

The right-hand side of the above inequality tends to zero as *k* → *∞*, and the left-hand side is independent of *k*; therefore *μ*(*φ* ^*g*^) = Δ*φ*. Thus, *R*⊆*Q* and *ζ* ≤ *J*, which implies *ζ* = *J*.

**Proposition 3**

The measure μ* in the set *Q*_*M*_ at which the functional *I* attains its maximum has the form

(32) $$\mu^{*}=\overset{M}{\underset{j=1}{\sum }}{\alpha^{*}}_{j}\delta \left(z_{j}^{*}\right),$$

where *α*_j_* ≥ 0, *z*_j_* ∈ *Ω*, and *δ*(*z*) is unitary atomic measure with the support being the singleton set {*z*_j_*}, characterized by *δ*(*z*)(*F*) = *F*(*z*), *z* ∈ *Ω*.

**Proof**

See [35].

Therefore, our attention is restricted to finding a measure in the form of (32), which maximizes the functional *I* and satisfies in *M* number of the constraints (26)–(28). Thus, by choosing the functions *φ*_*i*_, *i* = 1,2,…, *M*_1_, *ψ*_*k*_, *k* = 1,…, *M*_2_, and *ϑ*_*s*_, *s* = 1,…, *S*, the infinite dimensional problem (25)–(28) is approximated by the following finite dimensional nonlinear programming (NLP) problem:

(33) $$\begin{array}{c} \underset{\alpha_{j}\geq 0,\;z_{j}\in \Omega }{\text{Maximize}}\;\overset{M}{\underset{j=1}{\sum }}\alpha_{j}f_{0}\left(z_{j}\right) \end{array}$$

subject to

(34) $$\begin{array}{c} \overset{M}{\underset{j=1}{\sum }}\alpha_{j}\varphi_{i}^{g}\left(z_{j}\right)=\Delta \varphi_{i},\;i=1,\ldots ,M_{1}, \end{array}$$

(35) $$\begin{array}{c} \overset{M}{\underset{j=1}{\sum }}\alpha_{j}\psi_{k}^{\;n}\left(z_{j}\right)=0,\;k=1,\ldots ,M_{2},\;\;n=1,2,3,4, \end{array}$$

(36) $$\begin{array}{c} \overset{M}{\underset{j=1}{\sum }}\alpha_{j}\vartheta_{s}\left(z_{j}\right)=a_{\vartheta_{s}},\;s=1,\ldots ,S, \end{array}$$

where *M* = *M*_1_ + 4*M*_2_ + *S*. Clearly, (33)–(36) is an NLP problem with 2 *M* unknowns: *α*_*j*_ and *z*_*j*_, *j* = 1,…, *M*. One is interested in LP problem. The following proposition enables us to approximate the NLP problem (33)–(36) by a finite dimensional LP problem.

**Proposition 4**

Let *Ω*_*N*_ = {*y*_1_, *y*_2_,…, *y*_*N*_} be a countable dense subset of *Ω*. Given *ε* > 0, a measure *v* ∈ *M* ^+^(*Ω*) can be found such that:

(37) $$\begin{array}{c} \left|v\left(f_{0}\right)-\mu^{*}\left(f_{0}\right)\right|\leq \varepsilon ,\\ \left|v\left(\varphi_{i}^{g}\right)-\mu^{*}\left(\varphi_{i}^{g}\right)\right|\leq \varepsilon ,\;i=1,\ldots ,M_{1},\\ \left|v\left(\psi_{k}^{n}\right)-\mu^{*}\left(\psi_{k}^{n}\right)\right|\leq \varepsilon ,\;k=1,\ldots ,M_{2},\;\;n=1,2,3,4,\\ \left|v\left(\vartheta_{s}\right)-\mu^{*}\left(\vartheta_{s}\right)\right|\leq \varepsilon ,\;s=1,\ldots ,S, \end{array}$$

where the measure *v* has the form

(38) $$v=\overset{M}{\underset{j=1}{\sum }}\alpha_{j}^{*}\delta \left(y_{j}\right),$$

and the coefficients *α*_j_*, *j* = 1,…, *M*, are the same as optimal measure (32), and *y*_*j*_ ∈ *Ω*_*N*_, *j* = 1,…, *M*.

**Proof**

We rename the functions *f*_0_, *φ*_*i*_^*g*^'s, *ψ*_*k*_^*n*^'s, and *ϑ*_*s*_'s sequentially as *h*_*j*_, *j* = 1,2,…, *M* + 1. Then, for *j* = 1,…, *M* + 1,

(39) $$\begin{array}{cc} \left|\left(\mu^{*}-v\;\right)h_{j}\right| & =\left|\overset{M}{\underset{i=1}{\sum }}\alpha_{i}^{*}\left[h_{j}\left(z_{i}^{*}\right)-h_{j}\left(y_{i}\right)\right]\right|\\ & \leq \left(\overset{M}{\underset{i=1}{\sum }}\alpha_{i}^{*}\right)\underset{i,j}{\max\;}\;\left|h_{j}\left(z_{i}^{*}\right)-\;h_{j}\left(y_{i}\right)\right|. \end{array}$$

*h*_*j*_s are continuous. Therefore, max _*i*,*j*_  can be made less than *ε*/∑_*j*=1_^*M*^ *α*_j_* by choosing *y*_*i*_, *i* = 1,2,…, *M*, sufficiently near *z*_i_*.

For constructing a suitable set *Ω*_*N*_, which preserves the relation (6), *J* is divided to *S* subintervals as follows:

(40) $$\begin{array}{cc} J_{s}=\left[t_{0}+\frac{\left(s-1\right)\Delta T}{S-1},t_{0}+\frac{s\Delta T}{S-1}\right), & \\ s=1,2,\ldots ,S-1,\;J_{S}=\left[t_{l},t_{f}\right), & \end{array}\;$$

where *t*_*l*_ is a lower bound for optimal time *t*_*f*_, which can be obtained by using a search algorithm based on golden section [36] or *Fibonnaci* search method [37]. Let $\overset{̅}{S}$ be the largest number such that $J_{\overset{̅}{S}}\subseteq [t_{0},t_{0}+\eta ]$. Set $J^{1}={⋃}_{s=1}^{\overset{̅}{S}}J_{s}$, $J^{2}={⋃}_{s=\overset{̅}{S}+1}^{S}J_{s}$, *Ω* ^1^ = *J* ^1^ × *A* × *U*, and *Ω* ^2^ = *J* ^2^ × *A* × {0}. Moreover, the intervals *A*_*i*_ (*i* = 1,2, 3,4) and *U* are divided, respectively, into *n*_*i*_ and *m* subintervals. So, the sets *Ω* ^*i*^, *i* = 1,2, are partitioned into $N_{1}=\overset{̅}{S}n_{1}n_{2}n_{3}n_{4}m\;$ and $N_{2}=(S-\overset{̅}{S})n_{1}n_{2}n_{3}n_{4}$ cells, respectively. One point is chosen from each cell. In this way, we will have a grid of points, which are numbered sequentially as *y*_*j*_ = (*t*_*j*_, *x*_1_*j*__,…, *x*_4_*j*__, *u*_*j*_), *j* = 1,…, *N*, where *N* = *N*_1_ + *N*_2_.

 Therefore, according to (38), the NLP problem (33)–(36) is converted to the following LP problem:

(41) $$\begin{array}{c} \underset{\alpha_{j}\geq 0}{\text{Maximize}}\;\overset{M}{\underset{j=1}{\sum }}\alpha_{j}f_{0}\left(y_{j}\right) \end{array}$$

subject to

(42) $$\begin{array}{c} \overset{N}{\underset{j=1}{\sum }}\alpha_{j}\varphi_{i}^{g}\left(y_{j}\right)=\Delta \varphi_{i},\;i=1,\ldots ,M_{1}, \end{array}$$

(43) $$\begin{array}{c} \overset{N}{\underset{j=1}{\sum }}\alpha_{j}\psi_{k}^{\;n}\left(y_{j}\right)=0,\;k=1,\ldots ,M_{2},\;\;n=1,\;2,\;3\;,4, \end{array}$$

(44) $$\begin{array}{c} \overset{N}{\underset{j=1}{\sum }}\alpha_{j}\vartheta_{s}\left(y_{j}\right)=a_{\vartheta_{s}},\;s=1,\ldots ,S. \end{array}$$

Here, we discuss suitable total functions *φ*_*i*_s, *ψ*_*k*_s, and *ϑ*_*s*_s. The functions *φ*_*i*_s can be taken to be monomials of *t* and the components of the vector *x* as follows:

(45) $$t^{i}x_{2}^{j},\;x_{2}^{j}x_{h}^{i},\;i\in \left\{0,1\right\},\;\;j\in \left\{1,2,\;\ldots \right\},\;\;h\in \left\{1,3,4\right\}.$$

In addition, we choose some functions with compact support in the following form [36, 37]:

(46) $$\begin{array}{c} \psi_{2r-1}\left(t\right)=\{\begin{array}{cc} \sin\;\left(\frac{2\pi r\left(t-t_{0}\right)}{\Delta T}\right) & t\leq t_{l}\\ 0 & \text{otherwise}, \end{array}\\ \psi_{2r}\left(t\right)=\{\begin{array}{cc} 1-\cos\;\;\left(\frac{2\pi r\left(t-t_{0}\right)}{\Delta T}\right) & t\leq t_{l}\\ 0 & \;\text{otherwise}, \end{array} \end{array}$$

where *r* = 1, 2,… and Δ*T* = *t*_*l*_ − *t*_0_. Finally, the following functions are considered that are dependent on *t* only:

(47) $$\vartheta_{s}\left(t\right)=\{\begin{array}{cc} 1 & t\in J_{s}\\ 0 & \text{otherwise}, \end{array}$$

where *J*_*s*_, *s* = 1,…, *S*, are given by (40). These functions are used to construct the approximate piecewise constant control [35–37]. By the above definition of *ϑ*_*s*_, we consider *t*_*f*_ as an unknown variable in the constraints (44) which can be written as

(48) $$\begin{array}{c} \;\overset{\ell }{\underset{j=1}{\sum }}\alpha_{j}=\frac{\Delta T}{S-1}\\ \vdots \\ \overset{(S-1)\ell }{\underset{j=\left(S-2\right)\ell +1}{\sum }}\alpha_{j}=\frac{\Delta T}{S-1}\\ \overset{S\ell }{\underset{j=\left(S-1\right)\ell +1}{\sum }}\alpha_{j}=t_{f}-t_{l}, \end{array}$$

where *ℓ* = *N*/*S*. Of course, we need only to construct the control function *u*(·), since *x*(·) can be obtained by solving the ODEs (8). By using simplex method, a nonzero optimal solution *α*_*i*_1__*, *α*_*i*_2__*,…, *α*_*i*_k__*, *i*_1_ < *i*_2_ < ⋯<*i*_*k*_ of the LP problem (41)–(44) can be found where *k* cannot exceed the number of constraints, that is, *k* ≤ *M*_1_ + *M*_2_ + *S*. Setting *α*_*i*_0__* = *t*_0_, a piecewise control function *u*(·) approximating the optimal control is constructed based on these nonzero coefficients as follows [35, 36]:

(49) $$u\left(t\right)=\{\begin{array}{cc} u_{i_{j}} & t\in \left[\overset{j-1}{\underset{h=0}{\sum }}\alpha_{i_{h}}^{*},\overset{j}{\underset{h=0}{\sum }}\alpha_{i_{h}}^{*}\right)\\ 0 & \text{otherwise} \end{array},\;j=1,2,\ldots ,k,$$

where *u*_*i*_*j*__is the 6th component of *y*_*i*_*j*__.

To start the proposed method, one needs to have *t*_*l*_. Here, a bisection method is proposed to find the desired lower bound *t*_*l*_ for optimal time *t*_f_*. This algorithm has a simple structure and is started with a given upper bound *t*_*u*_, where it is assumed that the lower bound starts with *t*_*l*_ = 0. Assuming that *t*_f_*(*t*_*l*_) denotes the solution of LP problem (41)–(44) corresponding to the given lower bound *t*_*l*_, the bisection method is outlined as follows.

**Algorithm 1 (estimation of the lower bound *t*_*l*_)**

First, let *τ* = [*t*_*l*_, *t*_*u*_], where *t*_*l*_ = 0 and *t*_*u*_ is an upper bound for *t*_f_*.

**Step 1**

Let *a* = (*t*_*l*_ + *t*_*u*_)/2 and solve the corresponding LP problem to find *t*_f_*(*a*). If no feasible solution is found for the corresponding LP problem or *t*_f_*(*a*) = *a*, set *t*_*u*_ = *a*; else set *t*_*l*_ = *a*.

**Step 2**

If the length of the interval *τ* = [*t*_*l*_, *t*_*u*_] is small enough, then choose *t*_*l*_ as a good estimation for lower bound *t*_f_* else, go to Step 1.

## 5. Numerical Results

In this implementation, we set *M*_1_ = 10 and choose functions *φ*_*i*_, *i* = 1,2,…, *M*_1_, from *C*′(*B*) as follows:

(50) $$x_{1},x_{2},x_{3},x_{4},x_{2}^{2},x_{2}^{3},x_{1}x_{2},x_{3}x_{2},x_{4}x_{2},tx_{2}.$$

Furthermore, we set *S* = 13 and *M*_2_ = 6. Setting *u* = 0 in (8), we find that at *t*_0_ = 1642, *x*(*t*_0_) = (8.19,35,0.05,0.04). Model parameters are chosen as follows [17]:

(51) $$\begin{array}{c} I_{\overset{̅}{P}}=1.0,\;\beta =0.01,\;{\overset{̅}{\tau }}_{p}=0.2,\;{\overset{̅}{c}}_{p}=0,\;\tau_{p}=0.001,\\ c_{p}=20,\;\theta =0.02,\;\gamma =0.8,\;\varepsilon =0.154,\;I_{C}=0.2,\\ \alpha =0.3,\;v=3, \end{array}$$

and the following initial condition is used:

(52) x(0)=(5,100,0.0005,0).

**Figure 1:**
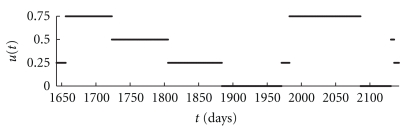
The approximate suboptimal piecewise constant control *u*.

**Figure 2:**
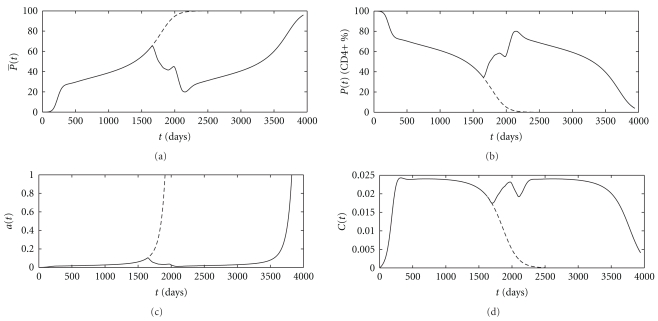
Dynamic behavior of the state variables $\overset{̅}{P}$, *P*, *a* and *C* versus time in the case of untreated (dashed line) and treated infected patients (solid line).

**Figure 3:**
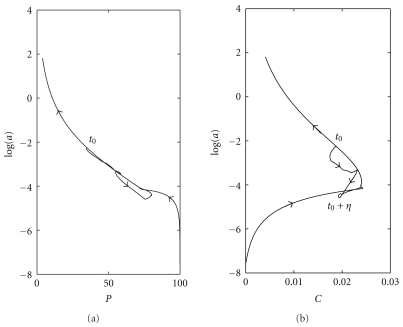
Phase space diagram for CD4+ T-cells (*P*), and CTLs (*C*).

Besides, *λ* is set to *λ* = 10, and the length of treatment is set to *η* = 500 (days). By using controllability on the dynamical control system, one can assume *A*_1_ = [7, 10], *A*_2_ = [5, 85], *A*_3_ = [0, 5], *A*_4_ = [0, 0.1], and *U* = [0,1]. Furthermore, the number of partitions in the construction of the set *Ω*_*N*_ are *n*_1_ = 4, *n*_2_ = 10, *n*_3_ = 4, *n*_4_ = 4, and *m*_1_ = 4. Initially, an upper bound for optimal time *t*_f_* is set to *t*_*u*_ = 5297.45. The results of implementing Algorithm 1 are summarized in Table 1. Setting *t*_*l*_ = 3642, we have an LP problem with 16000 unknowns and 47 constraints which is solved by the *linprog *code of the optimization toolbox in MATLAB. The total CPU time required on a laptop with CPU 2.20 GHz and 0.99 GB of RAM was 17.23 minutes. The suboptimal time has been found *t*_f_* = 3943.2. The resulting suboptimal control and the response of the system to the obtained control function are depicted in Figures 1 and 2, respectively. Moreover, we found *P*(*t*_f_*) = 4.9360, which is close to the exact value, that is, 5 (*CD*4_crit_^+^%). Note that the normal level of mature CD4+ T-cells is about 1000 cells/*μ*L. The relationships between the CD4+ T-cells, CTLs, and virus during the different stages of the disease are shown in Figure 3 as a phase space diagram.

## 6. Conclusion

 In this paper, we considered a dynamical system which describes the various aspects of the interaction of HIV with the immune system, to construct an optimal control problem which maximizes survival time of patients. A measure theoretical method is used to solve such kind of problems. The method is not iterative, and it does not need any initial guess of the solution, and numerical results confirmed the effectiveness of this approach. 

 Numerical results show that in presence of treatment, the survival time of patients can be considerably prolonged. From Figures 2(b) and 2(c), it is concluded that in presence of treatment (solid lines), the virus is controlled to very low levels and CD4+ T-cells are maintained at high levels for relatively long time. From Figure 2(d), an increase in CTL's occurs in response to therapy. 

Figure 3(a) shows an inverse correlation between CD4+ T-cells and virus particles. Furthermore, Figure 3(b) shows a clear correlation between the level of CTLs in the blood and HIV progression. As the virus increases upon initial infection, CTLs increase in order to decrease the virus. But this situation changes after about 1000th day due to destruction of CD4+ T-cells. Because these cells play an essential role in stimulation of immune response and signal other immune cells to eliminate infection by killing infected cells. After the 1642nd day, an increase in immune response can be observed which is due to recovery of CD4+ T-cells in response to treatment. Immune response increases for a while after discontinuation of therapy but ultimately becomes extinct.

## Figures and Tables

**Table 1:** Results of implementing Algorithm 1.

*t*_*l*_	*t*_*u*_	*a*	*t*_f_*(*a*)
0	5297.45	2648.72	3370.06
2648.72	5297.45	3973.08	Infeasible
2648.72	3973.08	3310.90	3595.25
3310.90	3973.08	3641.99	3709.73
3641.99	3973.08		
